# Children’s food allergy in Mexico

**DOI:** 10.1186/2045-7022-5-S3-P57

**Published:** 2015-03-30

**Authors:** Alejandra Medina-Hernandez, Ma del Carmen Zarate-Hernandez, Rosa Elena Huerta-Hernandez, David Alejandro Mendoza-Hernandez

**Affiliations:** 1 University of Queretaro, Queretaro, Mexico; 2 Hospital Universitario, Monterrey, Mexico; 3 Private Practice, Pachuca, Mexico; 4 National of Pediatrics Institute, Mexico City, Mexico; 5 Compedia, Colonia Nápoles, Mexico

## Background

MexiprevAAl is a prospective cross-sectional study carried out in 2000 patients recruited in outpatient clinics. Here we present results from pediatric population.

## Introduction

The aim of this nation-wide survey was to describe the profile of the patients with suspicion of food allergy seen by healthcare professionals, the normal clinical practice followed by the physicians, the social and healthcare repercussions of food allergy in Mexico.

## Methods

An observational, descriptive, cross sectional study was carried out from march 2013 to march 2014 using a convenience sample of allergic patients who were treated in departments, both private and public, of physicians who ween food alley patients.

## Results

Clinical, epidemiological, social data were collected from 1933 children with suspected food allergy. Preschool group was the most important followed over 14. The most suspected food was milk but the food changes with age.

## Discussion

Food allergy is highly suspected in children in Mexico, specially allergy due to milk. The food involved in food allergy changes with age. the clinical presentation changes with food, although the skin was the most affected organ. Food allergy has an important impact in quality of life of patients. Even the suspicious is high the use of diagnosis resources must be improved.

